# Husbands’ experience and perception of supporting their wives during childbirth in Tanzania

**DOI:** 10.1186/s12884-019-2715-7

**Published:** 2020-02-10

**Authors:** Denis Kampayana Kashaija, Lilian Teddy Mselle, Dickson Ally Mkoka

**Affiliations:** 1 Department of Obstetrics and Gynecology, SekouToure Regional Referral Hospital, PO Box 132, Mwanza, Tanzania; 2 Department of Clinical Nursing, Muhimbili University of Health and Allied Sciences, PO Box 65001, Dar es Salaam, Tanzania

**Keywords:** Husbands, Men, Support, Partner, Pregnancy, Labour, Delivery

## Abstract

**Background:**

In order improve the quality of birth care and women satisfaction with birthing process it is recommended that every woman should be offered the option to experience labour and childbirth with a companion of her choice. Involving husbands who are decision makers in the household may a play role in reducing maternal mortality which is unacceptably high despite the targeted goal to reduce this mortality up to three quarters as targeted in the MDGs by 2015. This is still addressed in the Sustainable Development Goals (SDGs) of 2015/30. This study aimed to explore the experiences and perceptions of husbands’ support of their wives during pregnancy, labour and deliveries in Tanzania.

**Methods:**

Qualitative descriptive study design was employed; involving men aged between 24 and 63 years. Participants were selected purposefully at the clinic and in labour ward of SekouToure Regional Referral Hospital (SRRH). The in-depth interview, guided by semi structured interview guide was used to collect the audio recorded and hand written information. Data were analysed using qualitative content analysis.

**Results:**

Nine semi-structured interviews were conducted with husbands of women attended for antenatal care and those came for deliveries. Four themes emerged; Demonstrating care, love and affection, adopting modern life style, observing women’s right and meeting social economic difficulties. Husbands’ support to their partners is a good behaviour practiced during matrimonial lives. Husbands who support their partners during pregnancy and delivery consider themselves as being modern men as they at home take duties beside their usual tasks to let their wives have adequate time to rest during pregnancy. Poor road infrastructure makes difficult to get transport to the healthcare facility especially when labour is imminent. Also ward infrastructure is not supportive to accommodate husbands when they accompany their wives to the healthcare facility.

**Conclusions:**

The healthcare settings in low income countries need to accommodate men during the routine antenatal and intranatal care for the positive outcome of labour and delivery.

Educating men on importance of active involvement in reproductive and child health services is important. Exploratory research should be conducted to understand how education and urbanisation affects men involvement in maternal and child health specifically in the low income countries.

## Background

Childbirth experience is a significant event in a woman’s life and is a powerful determinant of the use of maternal healthcare services [1] To improve quality of birth care and women satisfaction with birthing process the World Health Organization recommends that every woman is offered the option to experience labour and childbirth with a companion of her choice [2]. The Tanzania Ministry of Health has made strategies to ensure that each woman is accompaned during antenatal and delivery [3]. However, male companion during childbirth is still very rare as many women prefer a female companion who has at least given birth and is able to keep confidential information and is trusted by the woman [4].

In Tanzania as it is in other African countries husbands are head of families, control resources and commonly decide for their wives on where and when pregnant women should seek medical care even when their wives are economically well-off [4, 5]. Husbands are also social and economic powerful and they are traditionally seen by the community as facilitators of their wives’ access to reproductive health services [6, 7]. Studies in many countries have shown that involving men in reproductive health interventions can help improve maternal outcomes [7–10].

Male involvement in maternal and child health has been promoted for over a decade since the International Conference on Population and Development held in Cairo in 1994. However, many cultures in Africa and Asia have been considering pregnancy and childbirth and child rearing as woman’s responsibility [9]. Involving men in reproductive and child health issues has been a prominent part of the shift from family planning to the broader reproductive health agenda. Men obviously make up significant new customers for programs [10].

To promote quality maternal and child health care in Tanzania, various strategies were developed in 2018 including encouraging men to participate in reproductive and child health services. To ensure that men participate effectively and are involved right from the antenatal check-ups, the women were required to come to the clinic with their husbands for them to access services promptly [3]. The goal was to encourage husband to support their wives during labour and delivery and adequately prepare for birth and birth complications that may arise [7]. This strategy has shown significant impact on prevention of mother to child transmission of HIV program PMTCT ([11–13]; Report A. Sekoutoure RRH, Annual Hospital Report. Mwanza - Tanzania, unpublished). For example, a health facility based studies reported a prevalence of 70% in Tanzania, and 80% in Kenya of women accompanied by someone from their social network to the health facility during their childbirth [14, 15].

In spite of some improvement in involving men in reproductive and child health, little is known about the experiences and perceptions of men supporting their wives during labour and deliveries in Tanzania. Understanding experience and perceptions of husbands particularly on support they provided or thought they should provide may improve not only their involvement but also establish strategies that would foster husbands’ active participation during labour and delivery.

This study explored experiences and perceptions of support they provide to their wives during pregnancy, labour and deliveries among husbands who accompanied their wives to the health facility at SekouToure Regional Referral Hospital in Mwanza Region Tanzania. These findings intend not only form a platform for other studies in this study area, but also to enable policy makers to review the current strategy of male involvement in reproductive and child health using information from this study as evidence and consequently design strategies that improve services with reproductive health targeting male involvement.

## Methodology

### Study design, setting and participants

A descriptive research design [13] was used to explore experiences and perceptions of support from men who accompanied their wives during pregnancy, labour and deliveries. The study was conducted at SekouToure regional referral hospital (SRRH) in Mwanza Region. The hospital is a third referral level in the health system pyramid in Tanzania that offers a wide range of health care services including maternal and child health services.

It is a 320 total bed hospital with 71 beds dedicated for maternity services. About 30–50 deliveries occur each day according Annual hospital report – 2015 (Report A. Sekoutoure RRH, Annual Hospital Report. Mwanza - Tanzania, unpublished). Participants were purposeful recruited based on inclusion criteria. These criteria included; husbands who accompanied their wives for normal antenatal and delivery services, ability to speak Kiswahili and agree to participate in the study. The participants were recruited either after their wives have been assessed and admitted in the labour ward or after services have been provided at the antenatal or postnatal clinics. Other participants were recruited during the visiting hours when they came to visit their admitted wives. Participants were explained about the aim and benefits of the study. They were also told about the study procedure and the voluntary nature of their participation in the study and that they have the right to participate or to withdraw from the study at any time. All participants provided the written consent and the time and venue for interview was agreed.

### Data collection

The semi structured interview guide (Additional file 1) that was prepared by the researcher through review of literature and based on the experience of the researchers of conducting qualitative studies. The guide had open ended questions and probes related to experiences and perceptions of men who accompanied their partners/wives focusing on support that they provided and challenges encountered.

Nine semi structured interviews were conducted with husbands by the fist author (DKK) at the quiet suitable side room that was located within the maternity ward. To increase credibility of the findings, interviews were conducted in Kiswahili using semi structured interview guide and were audio recorded with participants’ permission. Interviews were recorded to ensure that description of the men’s experiences and perceptions on support they provide to their wives during childbirth is captured. Kiswahili language was used during interviews because is the language spoken fluently by both the participants and the researchers.. Every after each interview, the audio recorded interview was listened to and reflected on, and the guide was revised based on the new information obtained [13]. Interviews were conducted until when it was evident that there were no new information emerging in the interviews and previous shared information were repeating. Each semi-structured interview took approximately 20–35 min.

### Data analysis

Data was analysed based on content analysis framework [14]. The advantage of this analytical framework is that it is a concrete that could be readily applied and its ability to analyse data from the participants directly without imposing any other theoretical views by the researcher. The audio recorded interview voices were transcribed and translated from Kiswahili to English language by the first author. The co-authors reviewed the translated transcripts to ascertain the quality of translation [14, 15], and there were no significant differences between them. The second author (LTM) led the analysis process. The process began by reading and re-reading the transcripts to gain general understanding of the men’s experiences. Text, phrases and statements that describes men’s experiences when accompanying their wives to the hospital (meaning units) were extracted and condensed by shortening the original text, while maintaining the core meaning [14]. The meaning units were further condensed into codes that were sorted according to their similarities or differences into categories then the themes was obtained.

### Ethical considerations

Ethical approval was granted by the Research and Publication Committee of Muhimbili University of Health and Allied Sciences (MUHAS) (Ref. No. MU/PGS/SAEC/Vol.XIV) and Medical Officer Incharge of SekouToure Referral Regional (SRRH) gave permission to conduct the study. Participants provided written consent after they were explained the aim of the study, the procedure of data collection, issues of confidentiality, voluntary nature of participation and that they were free to withdraw their participation at any time, the decision that would not affect services to their wives. Further, oral permission was sought from participants on the use of audio-recorder during interview process.

## Results

During data analysis, a total of four main themes were identified relating to the experiences and perceptions of husbands who support their partners during pregnancy, labour and delivery. The identified themes were; demonstrating care, love and affection, men’s adoption with modern life style, observing women’s right and meeting socioeconomic difficulties during support. These themes together with corresponding categories are presented in Table 1 below.

**Table 1:** Themes and corresponding categories describing men’s experiences, perceptions and challenges of partner support during pregnancy labour and delivery

CATEGORIES	THEMES
Partner’s expectations of support Marriage commitment	Demonstrating care, love and affection
Men’s responsibilities in support and care during pregnancy, labour and delivery. Men’s preparations before delivery	Men’s adoption with modern life style
Community’s perspective on men’s support. Men’s expectations over support provided to partners.	Observing women’s right
Financial instabilities during the process of care provided by men. Constraints with transport for reaching the health care centres. Health care setting and the attitude of health care providers.	Meeting social economic difficulties

### Demonstrating care, love and affection

Participants interviewed in this study had different views about support they provide to their partners/wives during pregnancy, labour and delivery. They reported that support they provided to their partners was geared at ensuring the physical wellbeing of both mother and the coming baby.

“… *I make sure that she (the wife) gets proper diet during the daytime and during the night. All what I am doing is for the mother and for the coming baby…”* (Partner, 2).

Other participants shared that the support provided to their wives was appealing to what the couple sworn on the wedding day. It is a commitment which the two agreed as they married.

*“…as you swear during wedding you swear to be with your wife in happiness and problems because that was the agreement. So it is because of the agreement I made that I will be there to give her support…”* (Partner, 5).

Participants also thought that the support they provide has bases in their religious beliefs that they have to abide to in their denominations. Others thought of the relationship they have with their wives as the main reason to provide support:

“… *I see myself as the one who was involved to make her in this condition and if she succeeds it will be a gift to me, that is why I see it is important that we are together”* (Partner, 4).

Some participants were of the view that the provision of support to their partners during pregnancy or delivery was a matter of fulfilling’s women rights. Other participants insisted that caring their partners during pregnancy or delivery as more than women’s right but rather a human right:

*“… I believe I have the right to do so (to support my wife), it is all about women and their rights… I don’t feel at peace when I leave her alone. Because she knows there is somebody behind her and as I understand (we are one)”* (Partner, 5).

### Adopting modern lifestyle

Support provided by men was perceived by some participants as a way of conforming to a new life style as it was uncommon for husbands to accompany their wives during childbirth. It was however reported that for a long time men have been providing support to their partners, although not as it is now where provision of support to their partners is perceived as modern ways of life and moving with current era and increased levels of education among men:

*“…In the past men were supporting their wives partially, but because of development and education, people have identified the benefits of supporting their partners compared to previously when most men had low education...”* (Partner, 3).

Other participants had different views and argued that supporting partners during childbirth is not a matter of new fashion, but rather is an obligation as directed by religious teachings and according to the bible and that men cannot escape these responsibilities:

*“… No! It is not a fashion, to me I think it is normal, because even the writings in the word of God has insisted this, thus when you are two you need to assist each other as it is written in the Bible,…it is not good to leave your wife with problems without assisting…”* (Partner, 9).

Despite taking the role of a man or a husband in the family, participants reported specific responsibilities that need to be carried by the men when providing support to their wives during childbirth. This is the time when the usual duties of the woman at home are taken by her husband. Participants reported to practice what they were taught during antenatal care visits and therefore their support is not confined to reproductive health but it is beyond domestic chores:

*“… I am just supporting her when I have time in some of the days, to find food and helping home activities so that she can be kept free to make the unborn healthy. I took all the responsibilities at home including; washing, fetching water cleaning and mopping...”* (Partner, 8).

It was noted that some men were so keen to follow instructions given during antenatal visits especially on the issues to birth preparedness and that they were responsible for preparing all necessary requirements as needed, in response to the concept of individual birth preparedness, which is advocated during the routine clinic teaching. According to the participants, they were implementing what they gained from the sessions attended during the antenatal visits:

*“... I remember in the last visit we were told to be prepared for delivery, to have a safe place for delivery that she must have enough clothes, basin to go with; what to do about children at home I was not told, I just used experience. Her younger sister remained back at home to look after the young children. About money according to the jobs we are doing we don’t earn much so I just prepared with a little money for basic needs …”* (Partner, 2).

### Observing women’s rights

Husbands’ accounts indicated that men commonly supported their wives in favour of their rights. Community advocacy on women rights and encouraging men to observe these rights boosted the practice. Communities now perceive the support of men to their wives during childbirth as a normal event and need to be promoted among men. It was also learned that men are ready to accompany a woman in labour to the hospital if her husband is not around:

*“… they usually say that this man loves his wife, if men could be like this man, our marriages could be better….”* (Partner, 3).

*“… I think they see it normal, because as I informed about five people about what I was going to do … they took it as normal even”* (Partner, 4).

However, not all members of the community perceive that men have the role of supporting their wives during childbirth:

*“… it is not easy to know how others are thinking, there are others who are happy with what I am doing, and there are others wondering what happened to me. I see this as a normal thing”* (Partner, 4).

*“Every person has his own perspective; others may ignore or may perceive it as a normal issue according to one’s own culture …”* (Partner, 8).

It was also reported that the support provided by men was to ensure that the woman gets quick recovery so she can quickly get another pregnancy and thus increase number of children in the family. Others thought that the support they provide helps to prevent women from getting psychological problems and thus health of the unborn baby:

*“… Caring makes the woman to have no depression, because if you are not close to her she may have depression, and then you will ask why me, sometimes she may be bothered and this distraction is like being filled up with a certain poison, and this may affect the unborn baby. So it is all about making the future of the baby who will be born”* (Partner, 8).

### Meeting social economic difficulties

Participants reported that while making efforts to support their wives, they were confronted by some barriers that prohibited them from proving full support to their partners. These barriers included; financial instabilities, transport to the healthcare facility, attitude of the healthcare staff and ward environment. It was learned from this study that men had different occupations that make them have different levels of income and opportunity to accompany their partners to the healthcare facility. Participants were also concerned about the time they are needed to support their partner which compete with the time for generating income for their families. They reported that time spent in the caring of the partner affected the daily flow of money in their businesses. This was mentioned as the most constrain to full support their wives during pregnancy or delivery. Most participants in this study were either small scale businessmen or labourers. Some participants were concerned about the long time spent during ANC services or during delivery process. Others reported that time for ANC clinic for example coincided with open market, affecting their income generation:

“… *I usually have many activities those days that I am required to accompany my wife to the clinic. It is the market day however; I have go to the clinic with my wife. But then, sales and income from the market decreased …”* (Partner, 1).

Other participants reported that poor infrastructure increases time to arrive at the healthcare facility and thus not safe:

*“… Infrastructure is not friendly; I have changed the route so that I can reach here (healthcare facility). Roads are not good at all you will need a driver who is very professional with the right attitude…”* (Partner, 2).

Other participants reported that health workers were not welcoming, something that discouraged husbands from accompanying their wives. Husbands were prohibited to get inside the ward especially in the labour ward, denying their participation in the care of their wives in this process of labour and delivery. Additionally, health workers did not inform husbands of what was going on, leaving husband with the feelings of being abandoned and neglected. Participants however demonstrated desire to assist their wives during delivery process, however, such opportunity was not provided:

*“… I would like to assist my wife when struggling especially during pushing by holding her and encouraging her to push. She used to tell me that they use a lot of energy at this point and sometimes help can be available and sometimes there may be no help”* (Partner, 2).

*“I would like to witness the delivery of my baby, but because there is no possibility. Usually when you get here, they ask you to go out”* (Partner, 9).

It was reported by participants that they were not informed about what was going on with their wives when in the labour ward, they are just left alone waiting outside with lots of tension. They were concerned with the longer stay without getting feedback from health workers, for them this was dissatisfying and disappointing:

*“… I was received by the gate keeper, and told to go when my wife was in the ward. On arrival in the ward my wife was handed to a nurse on duty and I was told to go home without any more information. I was not satisfied as I expected to be asked to wait and be informed of the progress of my wife. My wife was not allowed to stay even with the phone.”* (Partner, 6).

## Discussions

In this study men’s support has been referred to as any care provided by a man to a pregnant woman and during delivery, including his physical presence either at home or at the health care facility. The study focused on men as their voice is overlooked in maternal health care also this is under research globally. Men involvement in maternal health care has been found as a new phenomenon in low resourced country as Tanzania, and the findings from this study may add up on the bank of information available in this country on men involvement in maternal health. Studies in Tanzania’s maternal health issues have been in the perspectives of either family planning issues or during maternal to child transmission (MTCT) of HIV as their main focus. The perspective of this study probably may change the focus on men’s support to their wives during pregnancy and the labouring process [11, 16, 17].

### Experiences and perceptions of men who support their partners during childbirth

Some informants in this study reported to have taken charge of home activities which women could be doing when pregnant including; cooking, mopping, washing, reminding their partners according to advices obtained during the clinic visit; like taking medications or vitamin tablets or Iron supplements, eat well, and do some exercises. In all these aspects, men are taking care of the home duties to relieve their partners, hence they have obeyed to theoretical focus of this study and the notion of masculinities (hegemonic notion of masculinity) [12, 17].

Men reported to be the overseer of everything at their homes, in parallel to this statement, it has been reported that Men tend to be the decision-makers within families and often take the lead in issues regarding the allocation of money, transport, women’s workload and access to health services, family planning and use of contraceptives [18].

Participants in this study also reported to be the overall supervisors in home related issues as their main responsibilities at the family level. For being an overseer to all home activities/affairs, this emphasizes a hegemonic masculinity ideal that men are the major decision makers in the home; therefore seem to carry authority than women [19].

Some husbands in this study wished to witness deliveries of their partner; therefore it is either agreeing in one way or contradicting in the other perspective. But we can still agree if we consider a study conducted in Ghana where Men also wanted to be part of the process and support their partners in their own ways. They wanted to see what happens to their wives and babies every day, and help them make it in this critical decision, the idea which is linked to protection of women during pregnancy and childbirth; that are vulnerable during this period and have to be protected [20].

Husbands in this study reported to have been received and handled at the clinics with priority because they were couples. These habits positively promote men involvement in maternal health and probably being given a priority, men feel motivated. When they reach the labour ward the story changes, because there is no space for men as they come with their partners and the handling is different from that at the clinic [16, 21].

Some husbands in this study reported to have taken charge of home activities which women could be doing during pregnant state, like the mentioned; cooking, mopping and even washing. Further more in the responsibilities these men mentioned on how they are responsible with reminding their partners according to advices obtained during the clinic visit. Example taking medications or vitamin tablets or Iron supplements, eat well, and some exercises. In all these aspects, despite that he is exercising fatherhood in the context of the ability to impregnate a woman, he is responding to the notion of masculinities. Furthermore, if the teaching at the clinic is effective, he is exercising the hegemonic notion of masculinity [19].

Husbands under this study stressed that the support provided is for strengthening marital relationship. Probably this is because of the pre-marital education given to couples in preparation for marriage, therefore men have to implement during marriage life. This reason can be linked with what was found in one of the descriptive reviews of male support during child birth, that one of the advantages identified was the improved sexual relationship among couples. If these teachings provided during pre-marital session are demonstrated as an essential, couples’ sexual and reproductive health may be improved probably even after pregnancy and delivery [21].

### Challenges encountered by men supporting their partners during childbirth

Of the challenges perceived by respondents, shortage of time to accompany the partner to the clinic was mentioned by informants during the interview in this study. This is probably may be the cause of insufficient support perceived by men who thought that if there could be adequate time they could have done better. This is also observed in other studies that men’s employment situations prevent them from their participation in antenatal clinic programs and even in the postnatal clinic participation [20, 24, 25].

In this study men reported to have obstacles when reached at the health care centre. At this point they either faced unpleasant welcome from the entrance by the gate keeper or improper instructions when they waited at the bench or outside [22]. The better way probably could be to have a good welcome from the entrance point and to get any feedback for what is going on after examination of their partners. This feedback is expected to come from a health care provider who received their partner at the clinic for a normal ANC care or in the labour ward for delivery. This was noted in a women cantered universal health coverage series; that barriers and challenges to male involvement exist at different societal and health system levels [23]. At the health service delivery level, challenges include; health providers’ attitudes, inadequate staff training, insufficient staff numbers, long waiting times, regulations in health care facilities, cultural and gender norms and men’s lack of knowledge regarding maternal and child health These are features common in some of the centres which provide ANC to pregnant women or during delivery [24].

In this study some husbands wished to witness their partners during the delivery process so that one could do something to their loved ones and probably they could feel the somewhat the same as their partners go through. One participant wanted to be there so that he could feed her partner. The challenge was that they are not allowed to get inside the delivery room at this setting. There was similar observation in a study done in Nepal, where husbands were invited to attend birth i.e. they wanted to be involved throughout pregnancy and birth after attending birth preparation classes. Similar observation was noted in a descriptive review which was looking into experiences of men who support their partners during delivery; it was found that men who had an opportunity to be with their partners during the delivery process expressed that they were happy to be present as their partners were going through pain [25].

Lack of space in the hospital setting and the labour ward in specific, had reduced the morale of the informants under this study towards their efforts on support. This probably is because even the space they used to meet their partners during the process of labour was so small and open in such that privacy of the two could not be observed. Contrary to this, the descriptive review done to evaluate experiences of men who support their partners during deliveries indicated lack of privacy as a concern because of inappropriate infrastructures of labour and birthing rooms. This was because most of the birthing rooms in the low resource setting are built in a traditional style where both audio and visual privacy is a challenge [25, 26].

### Methodological considerations and study limitations

This paper provides an insight of what are the experiences and perceptions of husbands who supported their wives during pregnancy and delivery and the challenges encountered during provision of such support as evidenced by quotes that support the presented findings. To ensure the findings are credible, participants were purposively selected to involve those husbands who escorted their partners during pregnancy or delivery who provided in depth information about the research questions under the study. Using expanded field notes that were recorded during data collection further increased credibility and dependability of the data. The authors are experienced midwives and researchers, therefore the findings emerged from analysis of collected data rather than on the researchers’ pre-existing understanding of the problem, this was done through multiple coding. Some limitations were observed despite the efforts to ensure the trustworthiness of the study. The study involved only men, it is likely that women could have different experiences and perceptions about support men provides during childbirth. However the study focused on men as their voice is overlooked and under researched globally. While this study provides important insights into men’s experiences and perceptions the risk of translation in the interpretation of the findings should be acknowledged. The analysis of the semi-structured interviews was completed in English from translated transcripts. However, the transcripts were verified by co-authors fluent in Kiswahili to ensure adequate translations and all codes and themes were discussed amongst the researchers by reviewing the original Kiswahili transcripts.

## Conclusions

Men have a significant position in providing support in maternal health through the roles identified as lived experiences and perception resulted from this study. Therefore recognizing the value of men’s roles will be an important step towards finding the solutions towards the challenges faced maternal health care in these settings.

Men involvement in maternal health care, especially during pregnancy has positive outcome of labour and probably afterwards. With this evidence there is a need to involve men from a pre-conception, also making a follow up probably after pregnancy and delivery more than in the current level. Improving the birthing places to accommodate men who wish to take part in birthing process as evidence has indicated of helpfulness for husbands and hence more respect for women. Early mass education so as to increase men’s knowledge and participation will be an important intervention.

From the perspective of this study, further research is recommended to explore on how urban variations affect male involvement in maternal and child health, and another study to quantify the level of knowledge among men on support provided to their partners in the similar settings.

## Supplementary information

**Additional file 1.** In-depth Interview Guide: Experience of men who support their partners during pregnancy, labour and deliveries.

## Data Availability

The datasets generated and analysed during this study are not publicly available since participants did not give consent for the public sharing of their information. However, summaries of the information are available from the corresponding author upon request.

## Abbreviations

ANC: Antenatal care
HIV: Human immunodeficiency virus
MTCT: Maternal to child transmission of HIV
MUHAS: Muhimbili University of Health and Allied Sciences
PMTCT: Prevention of mother to child transmission
SDG: Sustainable development goals
WHO: World Health Organization
