# Supplementary material for: Husbands’ experience and perception of supporting their wives during childbirth in Tanzania
Source: BMC Pregnancy Childbirth. 2020 Feb 10;20:85. doi: 10.1186/s12884-019-2715-7 (PMC7011545; doi:10.1186/s12884-019-2715-7)
Supplement: Supplementary file 1 — Additional file 1. In-depth Interview Guide: Experience of men who support their partners during pregnancy, labour and deliveries. [file 12884_2019_2715_MOESM1_ESM.docx]

**Additional Material 1:**

**In-depth Interview Guide: Experience of men who support their partners during pregnancy, labour and deliveries**

1. What does supporting your partner during childbirth mean to you? (Probe: Marital obligation, religious obligations, women rights)
2. What are the specific responsibilities of the men when supporting partners during pregnancy, labour and delivery? (Probe: Financial, emotional, physical)
3. How do you describe your experience/feelings when your partner began labour pains? (Probe: getting to the health facility, received in the health facility, privacy maintained, staff attitude, hospital environment)
4. What challenges do men encounter when supporting their partners during labour and delivery? (Probe: Financial, transport, getting information from the health facility staff)
5. What is your suggestion about the roles of men in supporting their wives during childbirth?
